# Postoperative femoral head cartilage injury after hip arthroscopic treatment for femoroacetabular impingement syndrome and labral tear

**DOI:** 10.1186/s10195-024-00811-0

**Published:** 2024-12-18

**Authors:** Guanying Gao, Yichuan Zhu, Siqi Zhang, Yingfang Ao, Jianquan Wang, Yan Xu

**Affiliations:** 1 Department of Sports Medicine, Peking University Third Hospital, Institute of Sports Medicine of Peking University, 49 North Garden Road, Haidian District, Beijing, 100191 China; 2 Beijing Key Laboratory of Sports Injuries, Beijing, China; 3 Engineering Research Center of Sports Trauma Treatment Technology and Devices, Ministry of Education, Beijing, China

**Keywords:** Hip arthroscopy, Cartilage injury, Femoroacetabular impingement syndrome, Labral tear

## Abstract

**Background:**

Postoperative femoral head cartilage injury (FHCI) is a rare condition that can be observed in a certain proportion of patients undergoing hip arthroscopy. However, the prevalence and associated factors of FHCI, and the effect of this condition on clinical outcomes still remain unknown.

**Patients and methods:**

Consecutive patients who were diagnosed with femoroacetabular impingement syndrome (FAIS) and labral tear and underwent hip arthroscopic treatment in our institute between July 2020 and July 2021 were retrospectively evaluated. Supine anteroposterior hip radiographs, cross-table lateral radiographs, magnetic resonance imaging (MRI), and computed tomography (CT) were obtained preoperatively. Postoperative MRI, at least 6 months after arthroscopy, was performed. Postoperative FHCI was evaluated by two surgeons through MRI. Preoperative patient-reported outcomes (PROs) including visual analog scale (VAS) for pain, and modified Harris Hip Score (mHHS) before surgery and at final followup were obtained.

**Results:**

A total of 196 patients were included. Postoperative FHCI was identified in 21 (10.7%) patients. The intraobserver reliability of the observer A and B for detecting postoperative FHCI using 3.0-T MRI was high (*k* = 0.929, and *k* = 0.947, respectively). The interobserver reliability between the two observers for detecting FHCI using 3.0-T MRI was high (*k* = 0.919). There was no significant difference in preoperative and postoperative mHHS, VAS, and percentage of patients who surpassed minimal clinically important difference (MCID) and achieved patient acceptable symptom state (PASS) between patients with and without postoperative FHCI (*P* > 0.05).

**Conclusion:**

Although postoperative FHCI was observed in 10.7% of patients, which was associated with larger labrum, this condition did not result in inferior clinical outcomes.

*Level of evidence* IV, retrospective case series.

*Trial registration* The Chinese Clinical Trial Registry approved the registration (ChiCTR2200061166). The date of registration is 2022-06-15.

## Introduction

Hip arthroscopy has developed rapidly in recent years and has been shown to significantly decrease pain and improve hip function in patients with femoroacetabular impingement syndrome (FAIS) and labral tear [1–4]. However, there are relatively few studies on imaging follow-up after hip arthroscopy [5]. According to the observation in our clinical practice, postoperative femoral head cartilage injury (FHCI) had been observed in a certain proportion of patients who underwent hip arthroscopy, which had been rarely reported. Postoperative, the linear chondral or osteochondral indentation on the anterior surface of the femoral head in parallel with the acetabular rim and labrum was considered to be FHCI. Such damage pattern was similar to the divot sign reported by Rosinsky et al. [6]. The femoral head divot sign was a rare arthroscopic finding during hip arthroscopy, and recognition of a femoral head divot may be valuable for the diagnosis of microinstability during hip arthroscopy and may help guide appropriate management [6]. The similar postoperative cartilage injury from a prominent anchor after shoulder arthroscopy had also been reported [7, 8]. However, the prevalence, associated factors, potential mechanism underlying FHCI, and its effect on clinical outcomes have not been reported in existing studies. Addressing this research gap may facilitate surgeons in identifying and avoiding postoperative FHCI in specific patients, thereby enhancing overall clinical outcomes.

The purpose of this study was to identify: (1) the proportion of postoperative FHCI following hip arthroscopy for treatment of FAIS and labral tear, (2) the associated factors with FHCI, and (3) the effect of FHCI on clinical outcomes. It was hypothesized that postoperative FHCI after hip arthroscopic treatment may be observed in a certain proportion of patients with specific factors related to microinstability, and FHCI may negatively influence clinical outcomes.

## Materials and methods

### Participants

After institutional review board approval from the ethics committee of Peking University Third Hospital (no. M2019193), consecutive patients who were diagnosed with FAIS and labral tear and underwent hip arthroscopic treatment in our institute between July 2020 and July 2021 were retrospectively reviewed. The inclusion criteria were as follows: patients who were diagnosed with (1) FAIS by clinical findings (persistent hip pain and positive physical examinations), plain radiographs (alpha angle > 55° and/or lateral center–edge angle (LCEA) > 40°), (2) labral tear by magnetic resonance imaging (MRI) [9, 10], (3) underwent hip arthroscopic treatment, and (4) had preoperative MRI and postoperative MRI at least 6 months following arthroscopy. The exclusion criteria were as follows: (1) patients with preoperative FHCI and (2) history of prior hip surgery. Informed consent was obtained from all participants.

### Surgical technique

All surgeries were performed by one senior surgeon (Y.X.) with over 10-years of experience in hip arthroscopy using a standard supine approach as described by Gao et al. [11]. In brief, a detailed inspection of the central compartment was performed to assess the acetabular rim, acetabular labrum, articular cartilage, and ligamentum teres after interportal capsulotomy. Labral repair, labral debridement, femoral osteoplasty, or acetabuloplasty was performed according to the intraoperative findings. Capsular closure was routinely done at the end of surgery.

### Data measurements

Supine anteroposterior hip radiographs, cross-table lateral radiographs, and CT were obtained for all patients preoperatively. Preoperative alpha angle and LCEA were measured as described by previous studies [12, 13].

**Fig. 1:**
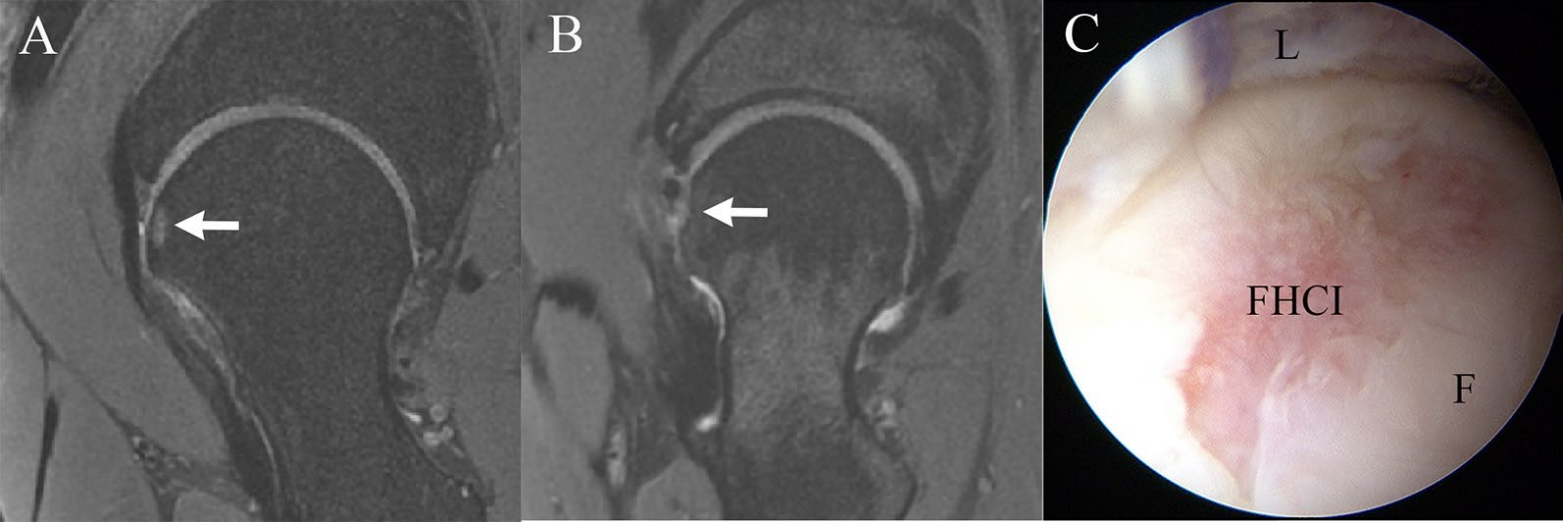
**A** Postoperative FHCI identified as concavity with a small area of hyperintensity of underlying bone observed on MRI. **B** Postoperative FHCI identified as a flattening of the head sphericity observed on MRI. **C** Postoperative FHCI observed in hip arthroscopy. White arrow, the area of postoperative femoral head cartilage injury. *FHCI* femoral head cartilage injury, *L* labrum, *F* femoral head

Patients underwent MRI preoperatively (the day before surgery) and at least 6 after arthroscopy (at clinic follow-up). As described by Gao et al. [14], the hip MRI was performed with a 3.0 T MR scanner (Magnetom Trio with TIM system, Siemens Healthcare) and a dedicated flexible surface coil around the affected hip joint. Fat-saturated proton density (FSPD) sequence and T2-weighted sequences were performed in the axial, coronal, and oblique sagittal planes respectively. Postoperative FHCI was identified as a flattening of the head sphericity or concavity with a small area of hyperintensity of the cartilage or underlying bone observed in the oblique sagittal or coronal plane on MRI (Fig. 1). Observer A with more than 5 years of experience with hip MRI and arthroscopy and observer B with more than 10 years of experience with hip MRI and arthroscopy analyzed all MRI scans. Any disagreements in findings were deferred to the corresponding author for final determination. The evaluations were performed twice by both surgeons to determine the intraobserver and interobserver reliability. The length of the acetabular hip labrum was measured at lateral and anterior anatomic sites along the acetabular rim as described by previous studies [15–17].

### Clinical outcomes

Preoperative and postoperative patient-reported outcomes (PROs) were obtained via questionnaires. PROs included visual analog scale (VAS) for pain and modified Harris Hip Score (mHHS). The PROs at final follow-up were evaluated at the same time as the MRI follow-up. For the 0–10 VAS, 0 means no pain and 10 means the worst pain. For the 0–100 mHHS, 0 means the poorest function and 100 means the most satisfied function. Complications or revision hip arthroscopy were recorded. For the mHHS, the minimal clinically important difference (MCID) was defined as 8 by Kemp et al. [18], and the patient acceptable symptom state (PASS) was defined as 74 by Chahal et al. [19].

### Statistical methods

Continuous variables with a normal distribution in the baseline data between groups were examined using the independent samples *t*-test. Mann–Whitney *U* test was used for non-normally distributed data. The two-tailed paired *t*-test was used to evaluate significance between preoperative and postoperative variables. Percentages were compared between patients with and without FHCI using the chi-squared test. The intraobserver and interobserver reliability was evaluated by calculating the kappa coefficient (*k*). *P* values < 0.05 were considered statistically significant. Confidence intervals (CIs) were set at 95%. All statistical analyses were performed with SPSS Statistics, version 22 (IBM).

## Results

### Participants

**Fig. 2:**
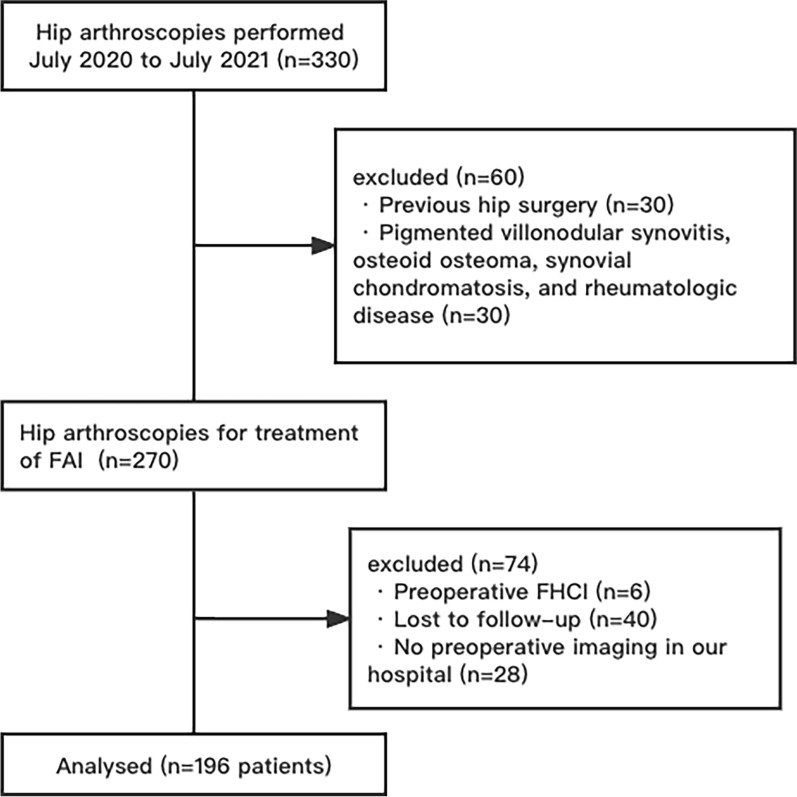
Flowchart illustrating patient selection process. *FHCI* femoral head cartilage injury

A total of 196 patients (mean age, 37.2 years; age range, 18–61 years; 76 male and 120 female) were included in this study. A flowchart illustrating the full patient selection process can be found in Fig. 2.

### Descriptive data

Alpha angle, LCEA, BMI, sides and diagnosis, and arthroscopic procedure are presented in Table 1. Among these 196 patients, 176 patients (89.8%) were diagnosed with cam-type FAI and 138 patients (70.4%) were diagnosed with pincer-type FAI; all patients had labral tear.

**Table 1:** Demography and arthroscopic procedures of patients (*n* = 196)

	Data
Age, years, mean (range)	37.2 (18–61)
Sex	
Male	76 (38.8%)
Female	120 (61.2%)
BMI, kg/m^2^, mean (range)	23.1 (16.2–31.1)
Alpha angle, degrees, mean ± SD	58.0 ± 5.9
LCEA, degrees, mean ± SD	34.3 ± 6.1
Mean follow-up time, months, mean (range)	26.3 (6–36)
Diagnosis	
Cam-type FAI	176 (89.8%)
Pincer-type FAI	138 (70.4%)
Labral tear	196 (100%)
Arthroscopic procedure	
Femoral osteoplasty	176 (89.8%)
Acetabuloplasty	138 (70.4%)
Labral repair	196 (100%)

Unless otherwise specified, data are numbers of patients, with percentages in parentheses

### Main results

The intraobserver reliability of the observer A for detecting postoperative FHCI using 3.0-T MRI was high (*k* = 0.929, 95% CI 0.874–0.981). The intraobserver reliability of the observer B was also high (*k* = 0.947, 95% CI 0.904–0.989). The interobserver reliability of the two observers for detecting FHCI using 3.0-T MRI was high (*k* = 0.919, 95% CI 0.864–0.971).

**Fig. 3:**
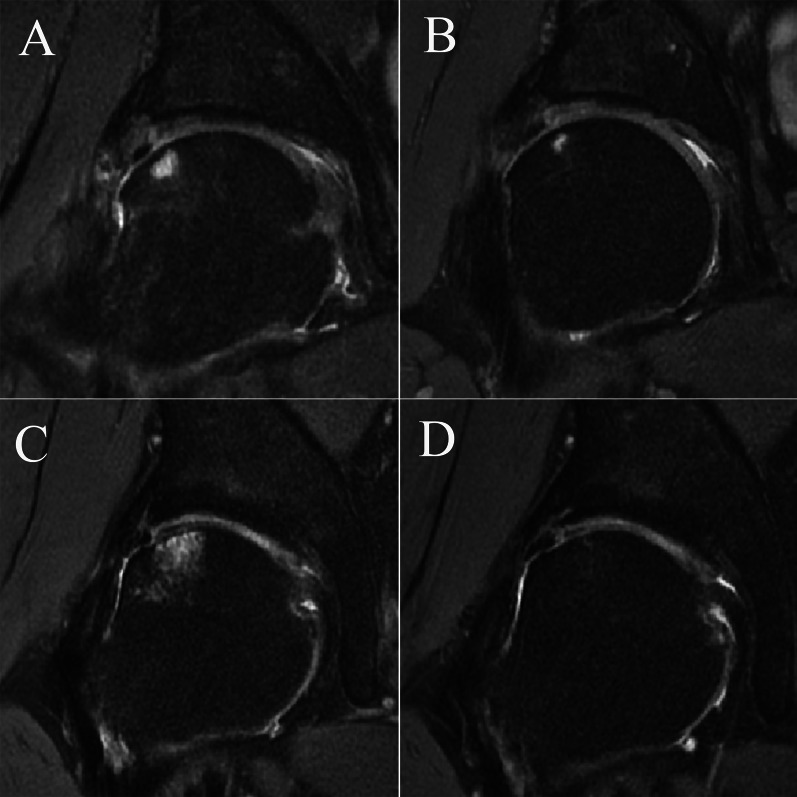
**A** Postoperative FHCI identified by MRI 1 year after hip arthroscopy. **B** The same patient underwent MRI again 6 months later, and the range of subchondral bone marrow edema of FHCI was significantly reduced. **C** Postoperative FHCI identified by MRI of another patient 1 year after hip arthroscopy. **D** Subchondral bone marrow edema of FHCI disappeared 6 months after the first MRI examination in this patient

Postoperative FHCI was identified in 21 (10.7%) patients. In patients with postoperative FHCI, no complaint of sensation of joint instability was reported. Mean postoperative LCEA in patients with postoperative FHCI was 34.3 ± 6.1°. There was no significant difference in postoperative LCEA and alpha angle between patients with and without postoperative FHCI (*P* > 0.05). In total, 2 of the 21 patients underwent further MRI follow-up 6 months after first MRI follow-up, and the range of subchondral bone marrow edema of FHCI was significantly reduced (Fig. 3).

As presented in Table 2, no significant difference was found in the demographic factors, proportion of diagnosis, and arthroscopic procedures between patients with and without FHCI (*P* > 0.05).

**Table 2:** Comparison of demography and arthroscopic procedures between patients with and without FHCI

	FHCI (*n* = 21)	No FHCI (*n* = 175)	*P* value
Age, years, mean (range)	37.2 (21–54)	37.1 (18–61)	0.96
Sex			
Male	7 (33.3%)	69 (39.4%)	0.58
Female	14 (66.7%)	106 (60.6%)	
BMI, kg/m^2^, mean (range)	23.9 (15.9–29.5)	23.0 (16.2–31.1)	0.26
Alpha angle, degrees, mean ± SD	58.0 ± 4.2	58.1 ± 7.4	0.97
LCEA, degrees, mean ± SD	33.0 ± 5.4	34.4 ± 7.4	0.41
Mean follow-up time, months, mean (range)	25.8 (6–33)	26.4 (6–36)	0.65
Diagnosis			
Cam-type FAI	18 (85.7%)	158 (90.3%)	0.51
Pincer-type FAI	14 (66.7%)	124 (70.9%)	0.69
Labral tear	21 (100%)	175 (100%)	> 0.99
Arthroscopic procedure			
Femoral osteoplasty	18 (85.7%)	158 (90.3%)	0.51
Acetabuloplasty	14 (66.7%)	124 (70.9%)	0.69
Labral repair	21 (100%)	175 (100%)	> 0.99

Unless otherwise specified, data are numbers of patients, with percentages in parentheses

In patients without postoperative FHCI, the mean preoperative lateral labral length and anterior labral length were 5.79 ± 2.09 mm and 7.59 ± 2.10 mm. In patients with postoperative FHCI, the mean preoperative lateral labral length and anterior labral length were 6.25 ± 1.70 mm and 8.48 ± 2.38 mm. Patients with postoperative FHCI had significant larger lateral and anterior labral size (*P* < 0.05).

Regarding PROs, both postoperative mHHS and VAS significantly improved from preoperative values in all patients (*P* < 0.05). As presented in Table 3, there was no significant difference in both preoperative and postoperative mHHS and VAS between patients with and without postoperative FHCI (*P* > 0.05). The percentage of all patients who achieved PASS was 94.3%. The percentage of patients who surpassed MCID was 84.3%. There was no significant difference in percentage of patients who surpassed MCID and achieved PASS between patients with and without postoperative FHCI (*P* > 0.05). No complications were reported. One patient (4.8%) in the FHCI group underwent revision arthroscopy, while no other patients reported undergoing revision surgery or conversion to total hip arthroplasty (THA).

**Table 3:** Comparison of PROs between patients with and without FHCI

	FHCI (*n* = 21)	No FHCI (*n* = 175)	*P* value
Preoperative VAS	4.6 ± 1.2	4.8 ± 1.6	0.68
Postoperative VAS	1.2 ± 0.8	1.0 ± 0.7	0.42
Preoperative mHHS	51.6 ± 8.3	48.6 ± 13.3	0.32
Postoperative mHHS	75.8 ± 17.1	78.1 ± 15.7	0.60
Achievement of MCID	18 (85.7%)	147 (84.0%)	0.89
Achievement of PASS	20 (95.2%)	165 (94.2%)	0.90

Data are presented as mean ± SD or numbers of patients, with percentages in parentheses

## Discussion

In this study, we found that postoperative FHCI was observed in 10.7% of patients after hip arthroscopic treatment for FAIS and labral tear. Patients with postoperative FHCI had significant preoperative larger lateral and anterior labral size. Postoperative FHCI may not have influence on the clinical outcomes after hip arthroscopy.

Rosinsky et al. [6] proposed the concept of divot sign to describe preoperative FHCI. They evaluated 690 patients and found 13 patients (2.0%) had evidence of a femoral head divot. Ligamentous laxity was present in 81.8% of those patients with divot sign, and the authors thought recognition of a femoral head divot may be valuable for the diagnosis of microinstability during hip arthroscopy and may help guide appropriate management. In our study, we excluded 6 (2.2%) patients with preoperative FHCI out of 270 patients who underwent hip arthroscopy for FAI. The percentage of patients with preoperative FHCI was similar to the percentage reported by Rosinsky et al. [6]. However, no evidence of microinstability was found in patients with postoperative FHCI. Although Rosinsky et al. proposed that microinstability may be the underlying reason for the FHCI, the number of patients with FHCI were relatively small. The exact reason of FHCI and the effect of this finding on patient outcomes are still uncertain.

In this study, we found that patients with postoperative FHCI had significant preoperative larger lateral and anterior labral size. The size of labrum was an important influential factor of postoperative FHCI. Previous studies have shown that chronic shear stresses between the femoral head and acetabular roof could result in compensational labral hypertrophy, which was a diagnostic criteria of microinstability in an international expert consensus [15, 20]. However, we considered larger labrum after suture may cause impingement between labrum and femoral head, leading to the generation of postoperative FHCI. Wang et al. [21] performed a biomechanical study that used silicone rubber to simulate an inverted acetabular labrum and put it between a hip model composed of hemispherical metallic platen (acetabulum) and a composite bone (femur). Then, the silicone rubber areas were subjected to extreme concentration of stress. Subchondral fractures of the femoral head developed at the silicone rubber areas. According to their study that suggested labrum may cause impingement on femoral head, we supposed that labrum after suture may also lead to impingement on femoral head, which caused postoperative FHCI. This also explained why larger labrum were more likely to cause postoperative femoral head cartilage or underlying bone damage. Further biomechanical studies are needed to explore the mechanism of postoperative FHCI.

It should be noted that there was no significant difference in both preoperative and postoperative PROs, as well as achievements of MCID and PASS between patients with and without postoperative FHCI. The influence of postoperative FHCI on clinical outcomes still need further studies with larger sample size and longer follow-up period. Meanwhile, 2 of the 21 patients with FHCI underwent further MRI follow-up 6 months after first MRI follow-up, and the range of subchondral bone marrow edema of FHCI was significantly reduced. Postoperative FHCI seems to gradually improve with recovery, and further study is needed to clarify the natural progression of postoperative FHCI.

## Limitations

This study had several limitations. First, the proportion of dropout was relatively high, which may cause risk of selection bias. Second, MRI diagnosis of postoperative FHCI has the possibility of misdiagnosis, though MRI diagnosis achieved high intraobserver and interobserver reliability. The gold standard for diagnosis of postoperative FHCI is hip arthroscopy, however only one patient underwent secondary arthroscopy in this study. Third, the number of patients with postoperative FHCI was relatively small, although it is the largest number, as far as we know. Further study was needed to identify the influencing factors.

## Conclusions

Although postoperative FHCI was observed in 10.7% of patients, which was associated with larger labrum, this condition did not result in inferior clinical outcomes.

## Data Availability

All relevant data supporting the conclusions are included within the article and tables. The datasets used and/or analyzed during the current study are available from the corresponding author on reasonable request.

## Abbreviations

FHCI: Femoral head cartilage injury
FAIS: Femoroacetabular impingement syndrome
MRI: Magnetic resonance imaging
CT: Computed tomography
PROs: Patient-reported outcomes
VAS: Visual analog scale
mHHS: Modified Harris Hip Score
MCID: Minimal clinically important difference
PASS: Patient acceptable symptom state
LCEA: Lateral center–edge angle
